# Meta‐analysis and GRADE profiles of exercise interventions for falls prevention in long‐term care facilities

**DOI:** 10.1111/jan.14238

**Published:** 2019-11-08

**Authors:** Daniela Schoberer, Helga E. Breimaier

**Affiliations:** ^1^ Institute of Nursing Science Medical University of Graz Graz Austria

**Keywords:** accidental falls, aged, exercise, GRADE approach, long‐term care, meta‐analysis, nursing, systematic review

## Abstract

**Aims:**

To provide a comprehensive collection of evidence on the effectiveness of exercise interventions to prevent falls and support clinical decision‐making.

**Design:**

A systematic literature review and meta‐analysis of randomized controlled trails were performed by combining trials from published systematic reviews and more recent ones from a separate literature search.

**Data Sources:**

The literature search was performed using PubMed, CINAHL, Cochrane Databases, and Google Scholar dating January 2007 – March 2018.

**Review Methods:**

Comparable studies were pooled using the random‐effects model. The GRADE approach was used to judge the evidence.

**Results:**

Exercises with a balance component or with technical devices reduced falls significantly, however, with low confidence in the evidence. The evidence indicated that exercises conducted longer than 6 months were beneficial. In frail residents, exercise interventions seemed to have substantially negative effects on falls.

**Conclusions:**

Exercise interventions with technical devices, those with a balance component, and those performed longer than 6 months are recommended in long‐term care settings. Frail residents need special attention when performing exercises due to their increased risk of falling.

**Impact:**

Exercises with a balance component and exercises carried out with technical devices have beneficial effects, whereas exercises performed longer than 6 months are more effective than short‐term performances. No significant reduction or decrease in the number of falls was found for exercise in cognitively impaired residents. In frail residents, however, the number of fall events increased substantially as a result of exercise interventions. The results of this review are useful for clinical decision‐makers in long‐term care facilities with regard to the planning and performance of exercise interventions for residents.

## Abstract (zh)

**目的:**

在于为预防跌倒和促进临床决策的运动干预有效性提供全面的证据收集。

**设计:**

通过结合已发表系统综述的试验和独立文献检索的近期试验,对随机对照试验进行系统文献综述和荟萃分析。

**资料来源:**

在PubMed、CINAHL、Cochrane Databases和Google Scholar上搜索了2007年1月至2018年3月的相关文献。

**综述方法:**

使用了随机效应模型来汇总了可比研究。采用了GRADE方法来判断证据。

**结果:**

减少使用平衡部件或技术设备的运动明显下降,但是对证据缺乏信心。有证据表明,锻炼时间超过6个月大有裨益。对于虚弱的居民,运动干预似乎对跌倒有很大的负面影响。

**结论:**

建议在长期护理环境中使用技术设备进行锻炼干预,使用平衡部件进行锻炼干预,以及采用时间超过6个月的锻炼干预。需要特别注意在进行锻炼时的虚弱居民,鉴于他们摔倒的风险与日俱增。

**影响:**

使用平衡部件的锻炼和使用技术设备进的锻炼同样大有裨益,而超过6个月的锻炼比短期锻炼更为有效。对于认知障碍类居民,没有发现因运动而导致跌倒次数显著减少或降低。然而,对于虚弱的居民,由于运动干预,跌倒事件的数量有所显著上升。本篇综述的结果能有助于长期护理机构的临床决策者规划和实施居民运动干预。

## 1 INTRODUCTION

Falls are still a global problem. It is estimated that about 600,000 falls with fatal consequences occur annually worldwide (World Health Organisation 2018). Older adults have an increased risk of falling. About half of all residents living in long‐term care facilities fall each year and one‐third suffer injuries that range from abrasions to traumatic brain injuries or hip fractures (Anderson, Boshier, & Hanna, 2012; Rapp, Becker, Cameron, Konig, & Buchele, 2012 et al. 2012). The estimated costs of care for those injured range from $1,750 to $22,130 per year (Heinrich, Rapp, Rissmann, Becker, & Konig, 2010). In addition to these physiological and economic burdens, falls can also lead to psychological consequences for the affected people, such as fear of falling (Kumar, Carpenter, Morris, Iliffe, & Kendrick, 2014). Furthermore, falls are also burdensome for nurses working in long‐term care facilities, as they experience feelings of guilt and feel as though they have failed (Bok, Pierce, Gies, & Steiner, 2016; Schoberer, Breimaier, Mandl, Halfens, & Lohrmann, 2016). Many nurses and residents attribute falls to the natural aging process and express the opinion that falls are inevitable and unavoidable (Schoberer et al., 2016). Despite this popular opinion, promoting the use of fall prevention interventions can potentially benefit residents living in long‐term care settings.

### 1.1 Background

Guidelines recommend multifactorial interventions with an exercise component for older people in long‐term care settings who are at risk of falling (National Institute for Health & Care Excellence, 2013). The effect of exercises as stand‐alone interventions in long‐term care facilities, however, is still not conclusive. Results from nine trials synthetized in a Cochrane review were inconsistent and showed positive effects of exercise interventions only for residents who lived in intermediate‐level care facilities (Cameron et al., 2014). A more recent meta‐analysis showed a beneficial effect for exercise interventions combined with other fall prevention interventions and exercise interventions with a balance component (Lee & Kim, 2017). Although several systematic reviews have been written on exercise interventions in long‐term care facilities, the conclusions are incongruent. These reviews had different foci (e.g., each placing a focus on one special type of exercise intervention) and used different inclusion criteria and different search strategies. Clinical decision‐makers, therefore, find it difficult to choose appropriate reviews that answer clinical questions that they consider as relevant and compare them with each other. A comprehensive review that addresses several relevant questions that arise in practice is needed.

## 2 THE REVIEW

### 2.1 Aims

This systematic review was conducted to provide a comprehensive summary of the randomized controlled trials (RCTs) that have dealt with exercise interventions and provide clear evidence that will facilitate decision‐making in clinical practice.

### 2.2 Design

We performed a systematic literature review and meta‐analysis of RCTs, by combining RCTs that have been included in published systematic reviews. This allowed us to identify a broad range of RCTs gathered using different search strategies and saved resources by not repeating the single RCT searches. More recently published RCTs were additionally obtained during a separate literature search. We used the GRADE approach to assess the evidence for exercise interventions with respect to fall prevention.

The studies included in this review were systematic reviews of RCTs or primary RCTs that dealt with any kind of exercise intervention, as a single intervention, in institutional long‐term care settings. This kind of setting included nursing homes, residential care facilities, homes for older people and assisted living facilities. Residents had to be older people, of whom more than 50% were older than 65 years of age or 70 years of age on average. The primary outcomes were the number of falls and fallers (those who had sustained a fall), whereas the secondary outcomes were the injuries sustained from falls, fear of falling, and quality of life. We excluded studies where multifactorial interventions with an exercise component were examined.

### 2.3 Search methods

The literature search was a two‐step process: Primarily, we conducted a search for systematic reviews of RCTs in the PubMed database, Cumulative Index of Nursing and Allied Health Literature (CINAHL) database, and the Cochrane Database of Systematic Reviews (CDSR) for the time frame of January 2007 – March 2018 written in English or German. Additionally, a manual search was conducted in Google Scholar and in reference lists of included reviews and of clinical guidelines (e.g., from the National Institute for Health & Care Excellence, 2013). To ensure that only systematic reviews with a low risk of bias were included, the reviews were appraised using the Critical Appraisal Worksheet for Systematic Reviews (Centre for Evidence‐based Medicine, 2005) and had to fulfil the following qualitative requirements: a clear description of the search methods, including a comprehensive literature search (at least two databases); a clear indication of inclusion and exclusion criteria; and a quality appraisal of included RCTs with predetermined quality criteria. Reviews were included either if they placed a sole focus on the effectiveness of exercise interventions for fall prevention or if they placed a focus on different kinds of fall prevention interventions, including exercise interventions as stand‐alone interventions to target only those studies.

After completing the systematic review search, we conducted a search for more up‐to‐date RCTs in the PubMed, CINAHL, and Cochrane Central Register of Controlled Trials (CENTRAL) databases as well as Google Scholar and reference lists. As the most recent comprehensive systematic review found had included studies that had been published in December 2014, the RCT search included the period of December 2014–March 2018. While conducting both searches, we used the same search terms (Search queries for systematic reviews, Table 1 in DataS1, Search queries for RCTs, Table 2 in DataS1), which were defined based on a preliminary literature search and discussions with experts in the field. The two authors checked the titles, abstracts and, subsequently, the full‐text articles according to the inclusion/exclusion criteria independently and discussed points of contention until a decision was made.

### 2.4 Search outcomes

**Figure 1:**
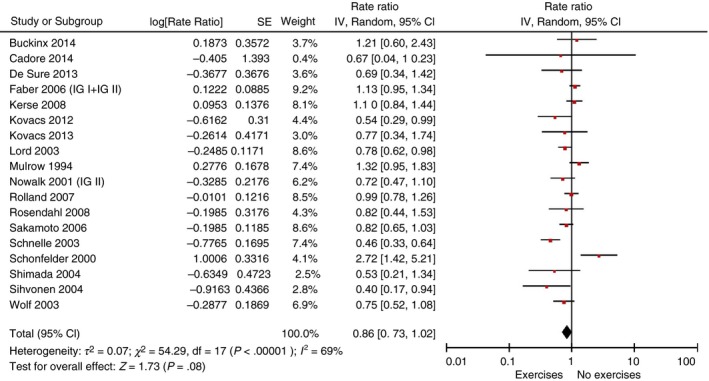
Forest plot of any kind of exercise intervention versus no exercise intervention, outcome rate of falls [Colour figure can be viewed at http://www.wileyonlinelibrary.com]

The database search yielded 112 reviews. Additionally, 13 reviews were identified through other sources. After removing duplicates and excluding records by carrying out title and abstract screening, we assessed 33 full‐text reviews. Nine of these reviews fulfilled the inclusion criteria (Cameron et al., 2014, Chan et al., 2015, Gleeson, Sherrington & Keay, 2014, Gregory & Watson, 2009, Harling & Simpson, 2008, Lee & Kim, 2017, Low, Ang, Goh, & Chew, 2009, Silva, Eslick & Duque, 2013, Vlaeyen et al., 2015) (PRISMA flow diagram, Figure 1 in DataS1). These reviews included 18 different RCTs that dealt with exercise interventions for fall prevention in long‐term care settings. The characteristics of the included systematic reviews, including the eligibility criteria used, search methods for the identification of studies as well as relevant RCTs included, are described in Supplement 2. The database search for recent RCTs yielded 95 records and three records were identified by examining additional sources. All five articles that were assessed for eligibility met the inclusion criteria (PRISMA flow diagram, Figure 2 in DataS1). Thus, the final synthesis included 23 RCTs.

### 2.5 Data abstraction

One author extracted the data using a predefined data extraction form and the other checked the accuracy of the extracted data. The following variables were extracted from each systematic review: eligibility criteria according to the population, interventions, outcomes and setting; and search methods including databases, additional sources, search periods, and restrictions (Supplement 2). In addition, we extracted data from each included RCTs that met the inclusion criteria defined above, according to the population, intervention details, results (relative and absolute data, if available), and quality appraisal (risk of bias). If data were missing from or incongruent between different systematic reviews, the original study was consulted. Similarly, data regarding the population, intervention, and results were extracted from the up‐to‐date RCTs.

### 2.6 Quality appraisal

The two authors independently appraised the methodological qualities of these RCTs using the Critical Appraisal Worksheet for Therapy Studies (Centre for Evidence‐Based Medicine, 2005). The critical appraisal questions and the corresponding categorisation regarding the selection, performance, detection, and attrition bias is provided in Table 2 in DataS3. Points of disagreement were resolved through discussion.

### 2.7 Synthesis

We grouped comparable studies with regard to the type, frequency, and duration of the intervention as well as special sample‐size characteristics. The synthesis was guided by the Cochrane Handbook (Higgins & Green, 2009). If at least two clinically comparable studies were available, these were pooled using the random‐effects model. This model is more likely to fit the sampling distribution and allows the results to be generalized to a wider array of situations (Borenstein, Hedges, Higgins, & Rothstein, 2010). We calculated pooled risk ratios for the dichotomous outcomes (e.g., fallers, fractures) and pooled rate ratios for the outcome rate of falls with 95% confidence intervals using the generic inverse variance method. Statistical heterogeneity was assessed using χ^2^ test (with statistically significance set at *p* < .05) and by calculating the *I^2^* statistic (Deeks, Higgins, & Altman, 2017). We used the Software Review Manager, Version 5.3 (Review Manager, 2014) to compile the meta‐analyses and calculate the heterogeneity. If the study results were presented in such a way that pooling was not possible, the *p* value is indicated. We performed sensitivity analyses to see if the overall estimate of effectiveness changed when the evidence from studies with a high risk of bias (at least two) was excluded (Higgins & Green, 2009) and at least 10 studies were identified.

To evaluate the confidence in the evidence of different kinds of exercise interventions, we used the GRADE method to evaluate each outcome. GRADE provides a framework that can be used to rate the quality of evidence and, thus, the confidence in estimates of the effects in a systematic, standardized manner (Balshem et al., 2011). In addition to the risk of bias (Guyatt, Oxman, Vist, Kunz, et al., 2011e), we evaluated the inconsistency of the results (Guyatt et al., 2011c), the indirectness in the applicability (Guyatt et al., 2011b), and the level of imprecision of the treatment‐effect estimate (Guyatt, Oxman, Kunz, Brozek, Alonso‐Coello, et al., 2011a) for each outcome. Starting from one of four evidence levels, the quality of evidence was graded one or two levels lower by the two authors while consulting with one another if one of the mentioned aspects was limited (e.g., high overall risk of bias or significant heterogeneity) (Balshem et al., 2011; Guyatt, Oxman, et al., 2013a). The lowest possible level given was one, which meant that little confidence was assigned the estimated effect (very low confidence in the evidence). In contrast, an evidence level of four meant that one could be highly confident that the true effect is similar to the estimated effect (high confidence in the evidence) (Balshem et al., 2011). The GRADE evidence profiles (Guyatt, Thorlund, et al., 2013b) that were finally developed during this process demonstrate the decisions transparently. The results of the meta‐analyses (pooled risk rate/rate ratio) or *p* values (if pooling was not possible) are as well incorporated into the GRADE evidence profiles (Tables 2, 3, 4).

## 3 RESULTS

### 3.1 Characteristics and quality of the included RCTs

The sample size ranged from 16–639 residents, with a total number of 3,767 participants. Performed exercise interventions were those to assess and improve balance, strength, walking, endurance, flexibility, functional, resistance, and mobility training with or without technical devices (e.g., vibration plate, Wii Balance Board). Five studies examined the effect of tai chi to prevent falls (Choi, Moon, & Song, 2005; Faber, Bosscher, Chin, & Wieringen, 2006; Nowalk, Prendergast, Bayles, D'Amico, & Colvin, 2001; Saravanakumar, Higgins, Riet, Marquez, & Sibbritt, 2014; Wolf et al., 2003). In four studies, more than one intervention arm was investigated (Faber et al., 2006; Nowalk et al., 2001; Saravanakumar et al., 2014; Tuunainen et al., 2013). Most studies had no exercise control group; three studies compared different exercise interventions with each other (Fu, Gao, Tung, Tsang, & Kwan, 2015; Sitjà‐Rabert, Martínez‐Zapata, M.a.J., Fort Vanmeerhaeghe, A., Rey Abella, F., Romero‐Rodríguez, D., & Bonfill, X., 2015; Tuunainen et al., 2013). Table 1 provides an overview of the study characteristics. All except one study (Choi et al., 2005) examined the outcome rate of falls, whereas 10 studies provided data for the outcome fallers (Buckinx et al., 2014; Choi et al., 2005; Faber et al., 2006; Kerse et al., 2008; Kovacs, Sztruhar Jonasne, Karoczi, Korpos, & Gondos, 2013; Mulrow et al., 1994; Rosendahl, Gustafson, Nordin, Lundin‐Olsson, & Nyberg, 2008; Sakamoto et al., 2006; Shimada, Obuchi, Furuna, & Suzuki, 2004; Sihvonen, Sipila, Taskinen, & Era, 2004). Additional, rarely investigated outcomes were hip fractures (Rosendahl et al., 2008), fear of falling (Wolf et al., 2003), and quality of life (Saravanakumar et al., 2014; Tuunainen et al., 2013). Table 1 in DataS3 includes a description of the quality of the included studies. The risk for selection bias was generally low and, on the contrary, several studies had a moderate to high risk of observer bias due to the lack of blinding or objective outcome measurements (Choi et al., 2005; DeSure, Peterson, Gianan, & Pang, 2013; Faber et al., 2006; Kerse et al., 2008; Kovacs et al., 2013, 2012; Lord et al., 2003; Mulrow et al., 1994; Nowalk et al., 2001; Rosendahl et al., 2008; Sakamoto et al., 2006; Schoenfelder, 2000; Shimada et al., 2004; Sihvonen et al., 2004).

**Table 1:** Characteristics of the included RCTs

Author, Country	Sample size (IG/CG)	Special sample characteristics	Type of exercise intervention	Control intervention	Intensity	Duration
Buckinx et al., 2014, Belgien	62 (31/31)		Whole body vibration (with vibration plate)	Usual care	3/week	6 months
Cadore et al., 2014, Spain	24 (11/13)	Frail according to Fried's criteria	Balance, strength	Routine mobility exercises	2/week	12 weeks
Choi et al., 2005 Korea	59 (29/30)		Tai chi	Usual care	3/week, 35 min	12 weeks
De Sure et al. 2013, USA	27 (12/15)		Balance, strength	Usual care	1/week	10 weeks
Faber et al., 2006, Netherlands	232 (64/78/90)^a^	Frail according to Fried's criteria	IG I: walking	Usual care	1–2/week, 60 min	20 weeks
			IG II: tai chi exercises, flexibility, balance, endurance, walking			
Fu et al., 2015, China	60 (30/30)		Balance (with Wii Balance Board)	Balance training (without balance board)	3/week, 60 min	6 weeks
Kerse et al., 2008, New Zealand	639 (310/329)		Functional (activity training with goal setting)	Usual care, social visits	Several times a day (short doses)	24 weeks
Kovacs et al., 2012, Hungary	41 (21/20)	Visual impairments	Balance, strength	Osteoporosis exercises only	2/week, 30 min	24 weeks
Kovacs et al., 2013, Hungary	166 (86/80)		Balance, strength	Usual care	2/week, 30 min	12 weeks
Lord et al., 2003, Australia	551 (280/271)		Balance, strength (group exercise)	Minimal‐intensity activities	2/week, 60 min	12 months
	SG 125 (64/61)	Cognitive impairments				
Mulrow et al., 1994, USA	194 (97/97)	Frail (depending in at least 2 ADL)	Gait, balance, coordination, functional, strength/resistance	Social visits	3/week, 30–45 min	16 weeks
Nowalk et al., 2001, USA	110 (38/37/35)^a^		IG I: tai chi	Basic enhanced programming	3/week	24 months
			IG II: strength, endurance			
Rolland et al., 2007, France	110 (56/54)	Alzheimer's disease	Balance, strength, flexibility, walking	Usual care	2/week, 60 min	12 months
Rosendahl et al., 2008, Sweden	183 (87/96)		Balance, strength (group exercise)	Activities performed while sitting (e.g., singing)	5/2 weeks, 45 min	3 months
	SG 97 (45/52)	Dementia				
Sakamoto et al., 2006, Japan	527 (315/212)		Balance (one leg‐standing)	Usual care	3/day, 2 min	24 weeks
Saravanakumar et al., 2014, Australia	33 (11/11/11)^a^		IG I: tai chi	Usual care	1/week, 30 min	14 weeks
			IG II: yoga			
Schnelle et al., 2003, USA	170 (85/85)		Strength, mobility, walking	Usual care	5/week	8 months
Schönfelder et al. 2000, USA	16 (9/7)		Balance, strength, walking	Usual care	3/week, 20 min	12 weeks
Shimada et al., 2004, Japan	26 (15/11)		Gait, balance, functional (with treadmill)	Usual exercises	3/week, 80 min	24 weeks
Sihvonen et al., 2004, Finland	27 (20/7)		Balance (on a force platform with a visual feedback screen)	Usual care	3/week, 20–30 min	4 weeks
Sitjà‐Rabert et al., 2015, Spain	159 (81/78)		Whole body vibration (with vibration plate)	Balance, strength training (without vibration)	3/week, 30 min	6 weeks
Tuuainen et al. 2013, Finland	55 (18/18/19)^a^		IG I: strength with physiotherapy	Self‐training with written instructions	2/week, 60 min	13 weeks
			IG II: strength, balance with physiotherapy			
Wolf et al., 2003, USA	296 (145/141)		Tai chi	Education	2/week, 90 min	48 weeks

Abbreviations: ATL, Activities of Daily Living; CG, Control Group; IG, Intervention Group; SG, Subgroup.

a 3 arms (IG I, IG II, CG)

### 3.2 Effects of exercise interventions for fall prevention

The results of the meta‐analysis of 18 RCTs (Figure 1; Figure 1.1 in DataS4) showed that performing any kind of exercise interventions compared with no exercise intervention had a non‐significant effect regarding the rate of falls (RR: 0.86, 95% CI [0.73–1.02]) with moderate statistical heterogeneity (*I^2^:* 69%). One study, which provided data on the outcome rate of falls in such a way that pooling was not possible, confirmed the non‐significant effect (Saravanakumar et al., 2014). Excluding the three studies with frail residents (Cadore et al., 2014; Faber et al., 2006; Mulrow et al., 1994) led to an identification of a significant effect for these exercise intervention (RR: 0.80, 95% CI [0.67–0.97], *I^2^:* 64%, Figure 1.3 in DataS4). The number of people sustaining a fall (outcome fallers) could not be decreased by providing exercise interventions (10 studies, RR: 1.04, 95% CI [0.91–1.19], *I^2^:* 11%, Figure 1.2 in DataS4). Excluding the studies with frail residents (Faber et al., 2006; Mulrow et al., 1994) also did not reduce the number of fallers (eight studies, RR: 0.99, 95% CI [0.86–1.14], *I^2^:* 0%, Figure 1.4 in DataS4). Figures 1.1 and 1.2 in DataS4 show the funnel plots for the main meta‐analyses.

### 3.3 Effects and GRADE profiles of different types of exercise interventions

We grouped different types of exercise interventions into multicomponent exercises, single‐component exercises, exercises with a balance component, exercises with tai chi, and exercises with technical devices and guided exercises. Neither multicomponent exercises nor single‐component exercises had significant effects on the rate of falls (RR: 0.82, 95% CI [0.66–1.03], *I^2^:* 73%, RR: 0.81, 95% CI [0.61–1.07], *I^2^:* 80%, Figures 2.1 and 3.1 in DataS4) with the confidence in the evidence graded as very low. The number of people sustaining a fall increased with multicomponent exercises as well as single‐component exercises (Figures 2.2 and 3.2 in DataS4, low confidence). No significant effect was found in one study for multicomponent exercises regarding reductions in hip fractures (Rosendahl et al., 2008). The confidence in this evidence was also very low due to the high risk of bias and broad confidence intervals. Exercises with a balance component reduced the number of falls significantly (RR: 0.79, 95% CI [0.65–0.98], *I^2^:* 68%, Figure 4.1 in DataS4), however, with low confidence in the evidence. Exercises with tai chi showed no effect compared with the no‐exercise control groups on the number of falls (Figure 5.1 in DataS4), fallers (Figure 5.2 in DataS4), or their quality of life with low and very low confidence in the evidence, but did show an effect on the fear of falling (*p* < .001) with moderate confidence. Exercises conducted with technical devices showed a significant, pooled effect (RR: 0.55, 95% CI [0.30–0.99], *I^2^:* 62%, Figure 6.1 in DataS4), but due to the moderate statistical heterogeneity, only low confidence in the evidence could be demonstrated. Removing the studies with whole body vibration (Buckinx et al., 2014; Sitjà‐Rabert et al., 2015) which caused the heterogeneity, the effect became higher (RR: 0.39, 95% CI [0.26–0.60], *I^2^:* 0%, Figure 6.3 in DataS4) and the confidence in the evidence increased to moderate. A beneficial effect for guided exercises as compared with self‐training with written instructions could be shown, but with very low confidence in the evidence (RR: 0.54, 95% CI [0.36–0.82]). The GRADE profile in Table 2 shows the quality assessment of the studies in detail and the results from the meta‐analyses with the confidence ratings. Detailed meta‐analyses with forest plots are shown in Figures 2.1–6.4 in DataS4. Study results that presented the data in such a way that pooling was not possible are listed separately in the GRADE profiles and the references for these studies are provided in the legend.

**Table 2:** GRADE profile: types of exercise interventions

No. of studies	Quality assessment				No. of participants		Pooled Risk Rate/Rate Ratio (95% CI)	Confidence in evidence
	Risk of Bias	Inconsistency	Indirectness	Imprecision	IG	CG		
Multicomponent exercises vs. no exercises								
Number of falls								
13	Serious^1^	Serious^2^	Not serious	Serious^3^	878	882	0.82 (0.66–1.03)	Very low
Fallers								
4	Serious^4^	Not serious	Not serious	Serious^3^	348	363	1.05 (0.87–1.25)	Low
Hip fractures								
1^a^	Serious^4^	Not serious	Not serious	Very serious^5^	87	96	0.16 (0.01–2.81)	Very low
Single‐component exercises vs. no exercises								
Number of falls								
8	Serious^4^	Very serious^6^	Not serious	Serious^3^	933	859	0.81 (0.61–1.07)	Very low
Fallers								
5	Serious^4^	Not serious	Not serious	Serious^3^	738	668	1.06 (0.87–1.29)	Low
Exercises with balance‐component vs. control								
Number of falls								
15	Serious^1^	Serious^2^	Not serious	Not serious	1,153	1,022	0.79 (0.65–0.98)	Low
Fallers								
7	Serious^1^	Not serious	Not serious	Serious^3^	698	593	0.98 (0.84–1.14)	Low
Exercises with tai chi vs. no exercises								
Number of falls								
2 (pooled) 2 (non‐pooled)^b^, ^c^	Serious^4^	Not serious	Not serious	Serious^3^	291	297	0.89 (0.71–1.11) *N*. sign. (*p* = .27, no *P*)	Low
Fallers								
2	Very serious^7^	Not serious	Not serious	Very serious^5^	107	120	1.03 (0.58–1.81)	Very low
Fear of falling								
1^d^	Not serious	Not serious	Not serious	Serious^8^	145	141	Sign. (*p* < .001)	Moderate
Quality of life								
1^c^	Serious^9^	Not serious	Not serious	Very serious^10^	22	11	*N*. sign. (no *P*)	Very low
Exercises with technical devices vs. control								
Number of falls								
4 (pooled) 1 (non‐pooled)^e^	Not serious	Serious^2^	Not serious	Serious^11^	177	157	0.55 (0.30–0.99) *N*. sign. (*p* = .41)	Low
Fallers								
3	Serious^4^	Not serious	Not serious	Serious^11^	66	49	0.79 (0.52–1.21)	Low
Exercises with technical devices vs. control (without whole body vibration)								
Number of falls								
3	Serious^4^	Not serious	Not serious	Not serious	65	48	0.39 (0.26–0.60)	Moderate
Fallers								
2	Serious^4^	Not serious	Not serious	Serious^11^	35	18	0.72 (0.43–1.19)	Low
Guided exercises vs. self‐training								
Rate of falls								
1^f^	Serious^f^	Not serious	Not serious	Very serious^f^	24	18	0.54 (0.36–0.82)	Very low
Quality of life								
1^f^	Serious^f^	Not serious	Not serious	Very serious^f^	24	18	*N*. sign. (*p *> .05)	Very low

Abbreviations: CI, Confidence Interval; CG, Control Group; IG, Intervention Group; *N*. sign, not significant; *P*, *P* value; sign, significant.

a Rosendahl et al. 2008.

b Nowalk et al. 2001.

c Saravanakumar et al. 2014.

d Wolf et al. 2003.

e Sitjá‐Rabert et al. 2015.

f Tuuainen et al. 2013.

^1^ High risk for detection and attrition bias.

^2^ Heterogeneity *(I^2^* > 50 < 75%).

^3^ CI overlaps no‐effect line.

^4^ High risk for detection bias.

^5^ Very broad CI.

^6^ Considerable heterogeneity *I^2^* > 75%.

^7^ High risk for detection, performance and attrition bias.

^8^ No sample size calculation for that outcome.

^9^ High risk for attrition bias.

^10^ Very small sample size (pilot study).

^11^ Broad CI.

### 3.4 Effects and GRADE profiles of exercise interventions differing in frequency and duration

Five studies (*N = *1,189) investigated exercise interventions with a duration longer than 6 months and 16 studies (*N = *2,255), with a duration of up to 6 months. The evidence indicated moderate confidence in the beneficial effect of exercises conducted longer than 6 months (RR: 0.73, 95% CI [0.57–0.94], *I^2^:* 71%, Figure 7.1 in DataS4). No data were available for the outcome fallers for this intervention. Exercises performed with a higher frequency, that is, three times or more often per week, showed no significant effect on the number of falls or fallers (RR: 0.78, 95% CI [0.58–1.05], *I^2^:* 82%, RR: 1.04, 95% CI [0.89–1.22], *I^2^:* 0%, Figures 9.1 and 9.2 in DataS4). However, the confidence in this evidence was low or very low. The pooled effect of exercise interventions from 10 studies, comparing those that were performed with a lower frequency (i.e., less than three times a week) with control interventions, showed a significant positive effect (RR: 0.78, 95% CI [0.63–0.97], *I^2^:* 55%, Figure 10.1 in DataS4), but the confidence in the evidence was graded as low (Table 3). Figures 7.1–10.2 in DataS4 show meta‐analyses and forest plots of all pooled results relating to Table 3.

**Table 3:** GRADE profile frequency/duration of exercise interventions

No. of studies	Quality assessment				No. of participants		Pooled Risk Rate/Rate Ratio (95% CI)	Confidence in evidence
	Risk of Bias	Inconsistency	Indirectness	Imprecision	IG	CG		
Exercises >6 months vs. control								
Number of falls								
5	Not serious	Serious^1^	Not serious	Not serious	603	586	0.73 (0.57–0.94)	Moderate
Exercises ≤6 months vs. control								
Number of falls								
14 (pooled) 2 (non‐pooled)^a^	Serious^2^	Serious^1^	Not serious	Serious^3^	1,211	1,044	0.83 (0.65–1.05) *N*. sign. (no *P*, *p* = .41)	Very low
Fallers								
10	Serious^4^	Not serious	Not serious	Serious^3^	1,132	983	1.04 (0.91–1.19)	Low
Exercises ≥3/week vs. control								
Number of falls								
11	Serious^2^	Very serious^5^	Not serious	Serious^3^	986	863	0.78 (0.58–1.05)	Very low
Fallers								
7	Serious^4^	Not serious	Not serious	Serious^3^	817	717	1.04 (0.89–1.22)	Low
Exercises <3/week vs. control								
Number of falls								
10	Serious^4^	Serious^1^	Not serious	Not serious	858	799	0.78 (0.63–0.97)	Low
Fallers								
3	Serious^2^	Serious^1^	Not serious	Serious^3^	315	266	1.05 (0.76–1.44)	Very low

Abbreviations: CI, Confidence Interval; CG, Control Group; IG, Intervention Group; *N*. sign, not significant; *P*, *p *value. Saravanakumar et al. 2014.

a Sitjá‐Rabert et al. 2015.

^1^ Heterogeneity (*I^2^* > 50 < 75%).

^2^ High risk for detection bias.

^3^ CI overlaps no‐effect line.

^4^ High risk for detection and attrition bias.

^5^ Considerable heterogeneity *I^2^* > 75%.

### 3.5 Effects and GRADE profiles of exercise interventions for special resident groups

Seven studies or subgroups of studies investigated exercise interventions in special resident groups. The effects of exercise interventions in residents with visual impairments were marginally non‐significant (RR: 0.54, 95% CI [0.30–1.00]). As this result was only derived from one study with a small sample size (Kovacs et al., 2012), the confidence in this evidence is very low. In frail residents (studies where residents were defined as frail), the confidence in the evidence was moderate for the substantially negative effect of exercise interventions on the number of falls (RR: 1.17, 95% CI [1.00–1.36], *I^2^:* 0%, Figure 11.1 in DataS4). Exercises in cognitively impaired residents did not demonstrate significant effects (RR: 0.72, 95% CI [0.48–1.07], *I^2^:* 81%, Figure 12.1 in DataS4). However, low confidence was assigned to this evidence (Table 4). Detailed meta‐analyses with forest plots for the results in Table 4 are shown in Figures 11.1–12.1 in DataS4.

**Table 4:** GRADE profile: exercise interventions for special resident groups

No. of studies	Quality assessment				No of participants		Pooled Risk Rate/ Rate Ratio (95% CI)	Confidence in evidence
	Risk of Bias	Inconsistency	Indirectness	Imprecision	IG	CG		
Exercises in residents with visual impairments vs. no exercises								
Number of falls								
1^a^	Serious^1^	Not serious	Not serious	Very serious^2^	21	20	RR = 0.54 (0.30–1.00)	Very low
Exercises in frail residents vs. no exercises								
Number of falls								
3	Serious^3^	Not serious	Not serious	Not serious	250	200	RR = 1.17 (1.00–1.36)	Moderate
Fallers								
2	Serious^3^	Not serious	Not serious	Serious^4^	239	187	RR = 1.25 (0.97–1.60)	Low
Exercises in cognitive impaired residents vs. no exercises								
Number of falls								
3	Serious^3^	Very serious^5^	Not serious	Serious^4^	165	167	RR = 0.72 (0.48–1.07)	Very low

Abbreviations: CI, Confidence Interval; CG, Control Group; IG, Intervention Group.

a Kovacs et al. 2012.

^1^ Risk for detection and attrition bias.

^2^ Broad CI, small sample size.

^3^ High risk for detection bias.

^4^ CI overlaps no‐effect line.

^5^ Considerable heterogeneity *I^2^* > 75%.

### 3.6 Sensitivity analyses

In general, the pooled effect sizes from the higher quality studies were similar to those from all included studies, also showing a comparable degree of heterogeneity. The rate of falls for multicomponent exercises merely decreased to a level of significance in the sensitivity analysis. However, this pooled effect size was still marginally not significant when all studies were included. The results of the sensitivity analyses are shown in Figure 1–5 in DataS5.

## 4 DISCUSSION

As in other systematic reviews, our review provides some evidence for the effectiveness of exercise interventions in older, long‐term care residents (Cameron et al. 2014, Lee & Kim, 2017), but only when studies with frail residents are excluded. However, it must be considered that frailty is probably under‐reported in the single studies while interpreting this result, as only a few studies assessed frailty and examined data of frail residents in particular. Exercise interventions carried out with frail residents even were shown to lead to an increased risk of falling. Frailty is a common characteristic of long‐term care residents (Bandeen‐Roche et al., 2015) and this characteristic is associated with low activity levels, reduced activation, and mobility disabilities (Bandeen‐Roche et al., 2015; Overbeek et al., 2018). These disabilities, combined with the offered exercise interventions, may have led to physical overload, reducing the effectiveness of the exercise interventions aimed at fall prevention. However, it is important for frail older adults to do exercises to reverse or mitigate frailty, preserve their quality of life, and restore independent functions (Batt, Tanji, & Borjesson, 2013). Therefore, healthcare professionals should take the increased risk for falling under consideration when planning exercise interventions with frail residents.

We could not identify a preference for a single component compared with multicomponent exercise interventions. A preference for multicomponent exercises was only found when studies with a high risk of bias were excluded (sensitivity analysis). It seems as though the balance component is crucial for the success of exercise interventions for fall prevention. In their systematic review, Lee and Kim (2017) also found significant beneficial effects for exercise interventions only if they focused on balance exercises. Exercise interventions, including balance training, can also have significant, positive effects on the functional status and cognitive performance of older people (Bouaziz et al., 2016). However, the confidence in these pieces of evidence is low to very low, which means that the true effect may differ substantially from the estimated effect. As more research is conducted, these results are likely to change (Guyatt, Oxman, et al., 2013a). One reason for the very low confidence in the evidence was the serious or extremely serious heterogeneity observed among the study results. This may be the result of pooling kinds of exercise interventions that are simply too diverse. Nevertheless, some abstraction is necessary when pooling study results and the analyses were based on predefined subgroups rather than on heterogeneity, as recommended (Vaziri et al., 2016). Another reason for the reduced level of confidence was the serious risk of bias and especially of detection bias, in several studies that were included. The risk of detection bias can be minimized by blinding (Guyatt, Oxman, Vist, Kunz, et al., 2011e); however, this was, due to the nature of the intervention and outcome, rarely possible.

Tai chi was found not to be effective for fall prevention, but low confidence was assigned to this result. Other published reviews have concluded that tai chi only has an effect in younger and more robust older adults (Gregory & Watson, 2009; Harling & Simpson, 2008; Low, Ang, Goh, & Chew, 2009). However, one high‐quality study showed that providing tai chi reduces the fear of falling, which led to moderate support for the evidence regarding this outcome. This study included residents in a transitional stage to frailty (residents who did not meet the frail and vigorous criteria), which may be the reason why the fear of falling could be decreased but not the rate of falls (Wolf et al., 2003). Fear of falling was not defined as an outcome in studies with other types of exercises where a focus was placed on fall prevention, although fear of falling plays an important role in fall prevention as predictor of further falls (Lavedan et al., 2018).

Furthermore, exercises with technical devices demonstrated a significant effect with moderate support for the evidence. These technical devices included a Wii Balance Board, a force platform with visual feedback, and a bilateral separated treadmill (Fu et al., 2015; Shimada et al., 2004; Sihvonen et al., 2004). More technical devices are steadily finding their way in long‐term care institutions. Vaziri et al. (2016) showed that technology for fall prevention is well‐accepted and rated as easily usable by older people. However, little evidence for the influence of these new technologies on the quality of life exists. The results of our study show that exercise interventions with a vibration plate did not reduce the number of falls significantly (Buckinx et al., 2014; Sitjà‐Rabert et al., 2015); instead, it even led to an increase in the number of falls in the study performed by Buckinx et al. (2014). The authors concluded that a single intervention might not have an influence on a multifactorial event such as falls. In another systematic review, in contrast, whole body vibration was found to improve functional mobility in older adults (Lam, Lau, Chung, & Pang, 2012).

The results of our study show that only exercise interventions with a duration longer than 6 months reduce falls significantly. Although these results are only based on five studies, this evidence has moderate support. An RCT that was published after the study period and, therefore, was not included in the study confirms this result (Hewitt, Goodall, Clemson, Henwood, & Refshauge, 2018). Furthermore, a recently published systematic review found that exercise interventions that are sufficiently long‐lasting have the potential to improve gait abilities, which are protective factors if a fall occurs, in nursing home residents (Arrieta, Rezola‐Pardo, Gil, Irazusta, & Rodriguez‐Larrad, 2018).

GRADE provided us with a useful method, which we used to effectively grade the evidence in our systematic review. It was especially relevant and helpful in that it allowed us to handle the heterogeneity and uncertainty present in study results. Clinical decision‐makers assign importance not only to the estimate of the effect but also the confidence in this estimate (Guyatt et al., 2008). This was transparently and consistently assessed for all outcomes and integrated in the study results. Thus, clinicians can attach importance to the implementation of effective interventions with moderate to high confidence levels. Where low to very low confidence was assigned to the evidence, more research is necessary.

During the search for systematic reviews and inclusion of RCTs, we noted some assets and limitations. While our literature search placed a focus on studies extracted from a limited number of databases over a limited timeframe, this method enabled us to include the most relevant databases, languages, recent publication years, and sources and helped us identify grey literature, thereby saving resources. The data extraction and bias assessment, however, due to the nature of the method used, placed a certain degree of trust in the expertise of these authors. In addition, we did not control the definitions/tools used by individual study authors to detect bias, although we presumed that this was heterogeneous among the reviews. We could use the GRADE tool, however, to focus specifically on the overall quality of studies with similar interventions and the same outcomes, which allowed us to neglect any inconsistencies that may have occurred during our quality appraisals. GRADE authors recommend suspecting a publication bias in addition to a risk of bias, imprecision, inconsistency, and indirectness when the available evidence is derived from several small studies, most of which have been commercially funded (Guyatt, Oxman, Montori, et al., 2011d). As no commercially funded interventions were investigated in this review, the publication bias was not included in the evidence profiles. However, this limits the confidence in the evidence, which might be lower, if we had evaluated publication bias by performing and interpreting funnel plots for all meta‐analyses. As in the Cochrane Reviews, we pooled data when at least two comparable studies were available. Pooling a small number of studies with the random‐effect model is discussed controversially and Borenstein, Hedges, Higgins, & Rothstein (2009) describe their concern that the estimate of the between‐study variance can be incorrect and that the confidence interval can provide a false sense of assurance if a small number of studies are pooled. However, in our study, the meta‐analyses with only two studies always had a low level of heterogenity and the small number of studies was considered in our GRADE profiles by lowering the confidence in the evidence.

While preparing this manuscript, the Cochrane review on fall prevention from 2014 was published in an updated version (Cameron et al., 2018). Although some results are similar and, therefore, offer more confirmation than the new results (e.g., the non‐significant effect of exercises on the rate of falls), several new pieces of knowledge were generated by our review. The Cochrane meta‐analyses included an investigation of institutions with different levels of care, and we focused on special resident groups. Furthermore, the analyses of the types of exercises differ. The other authors pooled, for example, two studies with additional balance exercises and found a significant reduction in the rate of falls, but the evidence was of low quality. As we included all studies with a balance component, we could pool 15 studies and achieved one level higher in the quality rating. Furthermore, our review provides evidence for exercises interventions with different frequency and duration, unlike the Cochrane review. This knowledge is particularly valuable for practitioners when planning their exercise interventions.

## 5 CONCLUSION

The results of our review are useful for clinical decision‐makers, helping them assess the performance of exercise interventions to prevent falls. We are moderately confident that exercises carried out with technical devices such as Wii Balance Boards have the potential to reduce falls significantly. Currently, there is no sufficient evidence for recommending tai chi for fall prevention, but these kinds of exercises can help residents decrease their fear of falling. To decrease the fear of falling, exercises with tai chi are recommended. Exercises with a balance component showed promising results, although only low support for the evidence is currently available. More studies on these exercises may reveal more promising results. Interventions performed longer than 6 months were shown to effectively reduce the numbers of falls and are recommended in long‐term care settings. Frail residents need special attention if they are included in exercise intervention programmes, as they are at increased risk of falling. In further RCTs, the characteristics of the sample (e.g., cognitive state, frailty) should be reported carefully and assessed with the tested instruments. This would allow meta‐analyses to be performed for special resident groups and would presumably reduce the heterogeneity of study results, which at the same time would strengthen the confidence in the evidence. Future studies should additionally focus on the endpoints fear of falling and quality of life.

## CONFLICT OF INTEREST

There are no conflicts of interests.

## AUTHOR CONTRIBUTIONS

DS, HEB: Made substantial contributions to conception and design, or acquisition of data, or analysis and interpretation of data; DS, HEB: Involved in drafting the manuscript or revising it critically for important intellectual content; DS, HEB: Given final approval of the version to be published. Each author should have participated sufficiently in the work to take public responsibility for appropriate portions of the content; DS, HEB: Agreed to be accountable for all aspects of the work in ensuring that questions related to the accuracy or integrity of any part of the work are appropriately investigated and resolved.
