# Supplementary material for: Meta‐analysis and GRADE profiles of exercise interventions for falls prevention in long‐term care facilities
Source: J Adv Nurs. 2019 Nov 8;76(1):121–34. doi: 10.1111/jan.14238 (PMC6972676; doi:10.1111/jan.14238)
Supplement: Supplementary file 1 [file JAN-76-121-s001.docx]

**Supplement 1: Search queries and PRISMA flow diagrams**

Table 1: Search queries for systematic reviews and hints

| **Queries (Systematic review search)** | **Reviews identified** |
| --- | --- |
| **PUBMED (Query)** | |
| (("Movement"[Mesh]) OR exercis* OR practic* OR training* OR physiotherap* OR motion OR gymnastic* OR exertion OR activit* OR workout OR danc* OR (tai chi) OR taiji OR (tai ji) OR (tai-ji) OR physical OR yoga)) AND (("Accidental Falls"[Mesh]) OR falls*) AND (("Residential Facilities"[Mesh]) OR (nursing home)) Filters: Systematic Reviews; Publication date from 2007/01/01 to 2018/03/31 | 46 |
| **CINAHL (Query)** | |
| ((MH "Movement") OR exercis* OR practic* OR training* OR physiotherap* OR motion OR gymnastic* OR exertion OR activit* OR workout OR danc* OR (tai chi) OR taiji OR (tai ji) OR tai-ji OR physical OR yoga)) AND (MH "Accidental Falls") AND ((MH "Residential Facilities") OR (nursing home)) Limiters - Published Date: 20070101-20180331; Publication Type: Systematic Review | 48 |
| **COCHRANE Database of Systematic Reviews** | |
| ((Move* or exercis* or practic* or training* or physiotherap* or motion or gymnastic* or exertion or activit* or workout or danc* or (tai chi) or taiji or (tai ji) or tai-ji or physical or yoga) and (fall*) and (Residential Facilit* or nursing home)).af. Custom Range 01/01/2007 to 31/03/2018 | 18 |
| **Google Scholar (first 10 pages)** | 7 |
| fall AND randomized controlled trial AND (nursing home OR residential facilities OR long-term care) AND (exercise OR training OR dance OR physiotherapy), Limit: 2007-2018 |  |
| **Reference lists (reviews and guidelines)** | 6 |

Table 2: Search queries for RCTs and hints

| **Queries (RCT search)** | **RCTs identified** |
| --- | --- |
| **PUBMED** | |
| (("Movement"[Mesh]) OR exercis* OR practic* OR training* OR physiotherap* OR motion OR gymnastic* OR exertion OR activit* OR workout OR danc* OR (tai chi) OR taiji OR (tai ji) OR (tai-ji) OR physical OR yoga)) AND ("Accidental Falls"[Mesh]) OR fall*) AND (("Residential Facilities"[Mesh]) OR (nursing home)) Filters: Randomized Controlled Trial; Publication date from 2014/12/01 to 2017/05/22; English; German | 39 |
| **CINAHL** | |
| ( (MH "Movement") OR exercis* OR practic* OR training* OR physiotherap* OR motion OR gymnastic* OR exertion OR activit* OR workout OR danc* OR (tai chi) OR taiji OR (tai ji) OR tai-ji OR physical OR yoga) ) AND ( (MH "Accidental Falls") OR fall*) AND ((MH "Residential Facilities") OR (nursing home)) Limiters - Published Date: 20141201-20170531; Publication Type: Randomized Controlled Trial; Language: English, German  Search modes - Find all my search terms | 21 |
| **CENTRAL via Ovid** | |
| ((Move* or exercis* or practic* or training* or physiotherap* or motion or gymnastic* or exertion or activit* or workout or danc* or tai chi or taiji or tai ji or tai-ji or physical or yoga) and (fall*) and (Residential Facilit* or nursing home)).af. limit to yr="2014 - 2018" limit to randomized controlled trial | 35 |
| **Google Scholar (first 10 pages)** | 2 |
| fall AND randomized controlled trial AND (nursing home OR residential facilities OR long-term care) AND (exercise OR training OR dance OR physiotherapy), Limit: 2014-2017 |  |
| **Reference lists (of RCTs)** | 1 |


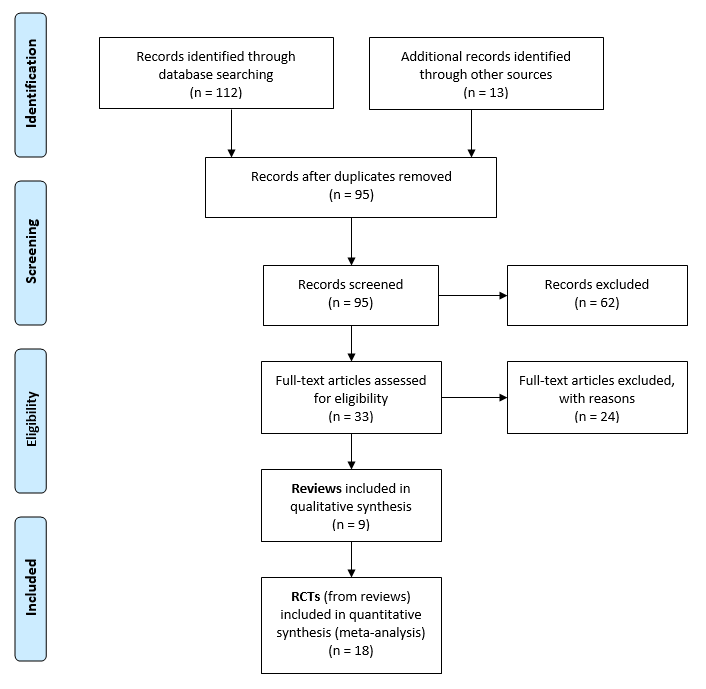


Figure 1: PRISMA flow diagram for systematic reviews


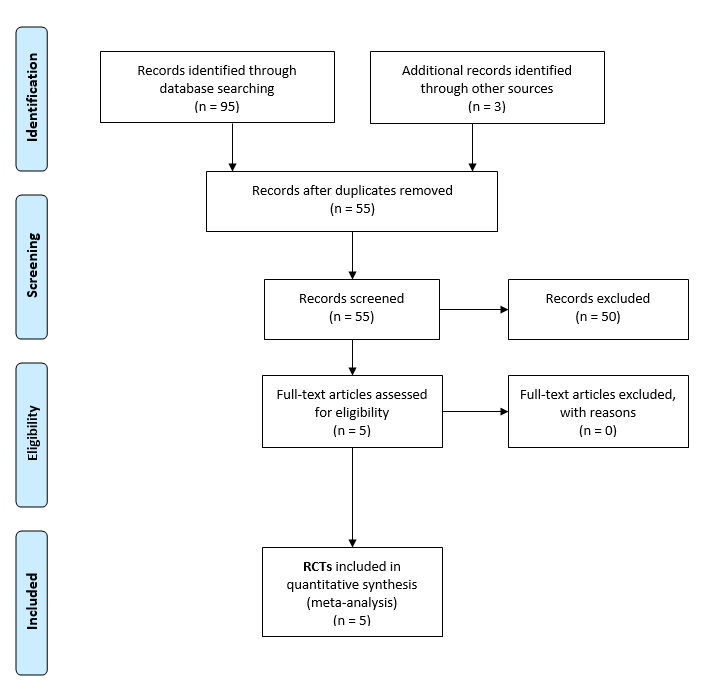


Figure 2: PRISMA flow diagram for RCTs
