# Supplementary material for: Meta‐analysis and GRADE profiles of exercise interventions for falls prevention in long‐term care facilities
Source: J Adv Nurs. 2019 Nov 8;76(1):121–34. doi: 10.1111/jan.14238 (PMC6972676; doi:10.1111/jan.14238)
Supplement: Supplementary file 2 [file JAN-76-121-s002.docx]

**Supplement 2: Characteristics of included systematic reviews**

| **Systematic Review authors** | **Eligibility criteria used according to** | | | | **Search methods for identification of studies** | | | | **Relevant RCTs** |
| --- | --- | --- | --- | --- | --- | --- | --- | --- | --- |
|  | **Population** | **Interventions** | **Outcomes (primary; secondary)** | **Setting** | **Databases** | **Additional sources** | **Time until** | **Language restriction** |  |
| Cameron et al. 2012 | Adults aged 65 years and older | Any intervention designed to reduce falls | Falls, fallers;  fractures, complication, costs | Residential care facilities, nursing care facilities or hospitals | Cochrane Specialized Register, CENTRAL, MEDLINE, Embase, CINAHL | WHO ICTRP, hand search in reference lists,  researchers contact | March 2012 | No language restriction | Choi et al. 2005, Faber et al. 2006, Kerse et al. 2008, Mulrow et al. 1994, Rosendahl et al. 2008, Sakamoto et al. 2006, Schönfelder et al. 2000, Shimada et al. 2004, Sihvonen et al. 2004 |
| Chan et al. 2015 | Oder adults with cognitive impairments | Physical exercise interventions | Falls;  fractures | All settings | MEDLINE, Embase, PsycINFO, CINAHL, CENTRAL, Cochrane Specialized Register, ClinicalTrials.gov | UK Clinical Research Network Study Portfolio | July 2013 | No language restriction | Lord et al. 2003, Rolland et al. 2007, Rosendahl et al. 2008 |
| Gleeson, Sherrington & Keay 2014 | Adults aged 60 years and older with untreatable visual impairments | Exercise or physical training | Physical function, falls | All settings | MEDLINE, Embase, CINAHL, CENTRAL, PEDro, LILACS | WHO ICTRP | February 2013 | No language restriction | Kovacs et al. 2012 |
| Gregory & Watson 2009 | Adults aged 60 years and older | Tai chi, exercise interventions with principles of tai chi | Falls | All settings | AMED, British Nursing Index, CENTRAL, CINAHL, Embase, MEDLINE, SPORTDiscus, Web of Knowledge | Hand search in reference lists | ~ 2008 (not indicated) | Not indicated | Faber et al. 2006, Nowalk et al. 2001, Wolf et al. 2003 |
| Harling & Simpson 2008 | Adults aged 60 years and older | Tai chi (as single intervention or one of a group of interventions) | Falls, fear of falling | Community or institutional care | MEDLINE, AMED, CINAHL, CENTRAL, PubMed, PEDro, Scirus | Hand search in reference lists | ~ 2007 (not indicated) | Not indicated | Nowalk et al. 2001, Wolf et al. 2003 |
| Lee & Kim 2017 | Adults aged 65 years and older | Any exercise intervention | Falls, fallers | Care facilities | MEDLINE, Embase, CINAHL, Cochrane Library, Korean databases (KoreaMed, KMbase, KISS, RISS, KisTi) | - | December 2014 | English, Korean | Cadore et al. 2014, Choi et al. 2005, De Sure et al. 2013, Faber et al. 2006, Kerse et al. 2008, Kovacs et al. 2012, Kovacs et al. 2013, Lord et al. 2003, Mulrow et al. 1994, Rosendahl et al. 2008, Sakamoto et al. 2006, Schönfelder et al. 2000, Shimada et al. 2004, Sihvonen et al. 2004, Wolf et al. 2003 |
| Low et al. 2009 | Adults aged 60 years and older | Tai chi | Falls | All settings | MEDLINE, Embase, Cochrane Library, Web of Science, CAB Direct | Hand search in reference lists, Google Scholar, hand search in JAGS | 2007 | English | Faber et al. 2006, Nowalk et al. 2001, Wolf et al. 2003 |
| Silva, Eslick & Duque 2013 | Adults aged 60 years and older | Physical exercises | Falls, fractures | Long term care facility | MEDLINE, Embase, PubMed, CENTRAL, PEDro, AMED, Occupational Therapy Seeker | Hand search in reference lists | Until June 2012 | English | Faber et al. 2006, Kerse et al. 2008, Lord et al. 2003, Nowalk et al. 2001, Rosendahl et al. 2008, Schnelle et al. 2003, Sihvonen et al. 2004, Wolf et al. 2003 |
| Vlaeyen et al. 2015 | Nursing home residents | Any fall prevention program with a duration of minimum 6 month | Falls, fallers, recurrent fallers | Nursing homes | MEDLINE, Embase, CENTRAL, PEDro, CINAHL, SPORTDiscus | Hand search in reference lists | September 2013 | English, French, German, Dutch | Schönfelder et al. 2000 |
| Abbreviations: AMED=Allied and Complementary Medicine Database, CINAHL=Cumulative Index of Nursing and Allied Healthcare Library, CENTRAL=Cochrane Central Register of Controlled Trials, LILACS=Literatura Latino-Americana e do Caribe em Ciências da Saúde, MEDLINE=Medical Literature Analysis and Retrieval System Online, PEDro=Physiotherapy Evidence Database, WHO ICTRP= World Health Organisation International Clinical Trials Registry Platform | | | | | | | | | |
