# Supplementary material for: Meta‐analysis and GRADE profiles of exercise interventions for falls prevention in long‐term care facilities
Source: J Adv Nurs. 2019 Nov 8;76(1):121–34. doi: 10.1111/jan.14238 (PMC6972676; doi:10.1111/jan.14238)
Supplement: Supplementary file 3 [file JAN-76-121-s003.docx]

**Supplement 3: Risk of bias in the included RCTs and corresponding questions**

Table 1: Risk of bias in the included RCTs

|  | Selektion bias | Performance bias | Attrition bias | Detection bias |
| --- | --- | --- | --- | --- |
| Buckinx et al. 2014 | **+** | **+** | **+** | **+** |
| Cadore et al. 2014 | **+** | **+** | **~** | **+** |
| Choi et al. 2005 | **-** | **-** | **~** | **-** |
| De Sure et al. 2013 | **+** | **-** | **-** | **~** |
| Faber et al. 2006 | **+** | **~** | **+** | **-** |
| Fu et al. 2015 | **+** | **+** | **+** | **+** |
| Kerse et al. 2008 | **+** | **~** | **+** | **-** |
| Kovacs et al. 2012 | **+** | **+** | **~** | **~** |
| Kovacs et al. 2013 | **+** | **+** | **~** | **~** |
| Lord et al. 2003 | **+** | **~** | **-** | **-** |
| Mulrow et al. 1994 | **+** | **+** | **~** | **~** |
| Nowalk et al. 2001 | **+** | **~** | **+** | **~** |
| Rolland et al. 2007 | **+** | **~** | **+** | **+** |
| Rosendahl et al. 2008 | **+** | **+** | **~** | **-** |
| Sakamoto et al. 2006 | **+** | **+** | **-** | **-** |
| Saravanakumar et al. 2014 | **+** | **+** | **-** | **+** |
| Schnelle et al. 2003 | **+** | **+** | **~** | **+** |
| Schönfelder et al. 2000 | **~** | **-** | **+** | **-** |
| Shimada et al. 2004 | **~** | **+** | **~** | **-** |
| Sihvonen et al. 2004 | **+** | **+** | **~** | **-** |
| Sitjà-Rabert et al. 2015 | **+** | **+** | **+** | **+** |
| Tuuainen et al. 2013 | **~** | **+** | **-** | **+** |
| Wolf et al. 2003 | **+** | **+** | **+** | **+** |

Explanation: + low risk, ~ moderate risk, - high risk

Table 2: Risk of Bias and corresponding questions according to the Critical Appraisal Worksheet for Therapy Studies (Centre for Evidence-Based Medicine 2005)

| SELEKTIONSBIAS |
| --- |
| Was the assignment of patients to treatments randomised? |
| Were the groups similar at the start of the trial? |
| PERFORMANCEBIAS |
| Aside from the allocated treatment, were groups treated equally? |
| ATTRITIONBIAS |
| Were all patients who entered the trial accounted for? – and were they analysed in the groups to which they were randomised? |
| OBSERVERBIAS |
| Were measures objective or were the patients and clinicians kept “blind” to which treatment was being received? |
