# Supplementary figures and images for: Meta‐analysis and GRADE profiles of exercise interventions for falls prevention in long‐term care facilities
Source: J Adv Nurs. 2019 Nov 8;76(1):121–34. doi: 10.1111/jan.14238 (PMC6972676; doi:10.1111/jan.14238)

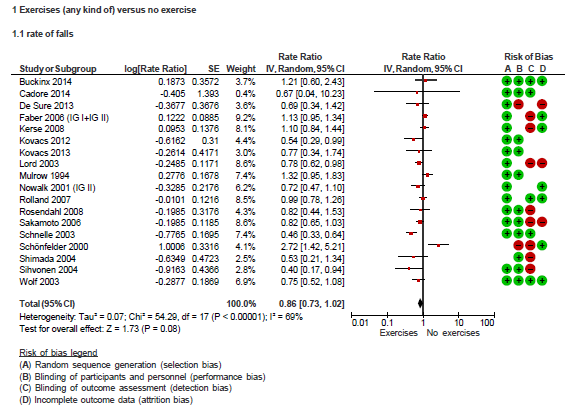
Supplement 4: Forrest Plots of all analyses and selected Funnel Plots


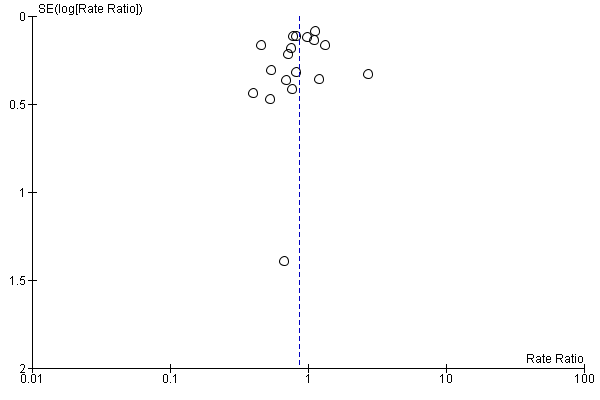


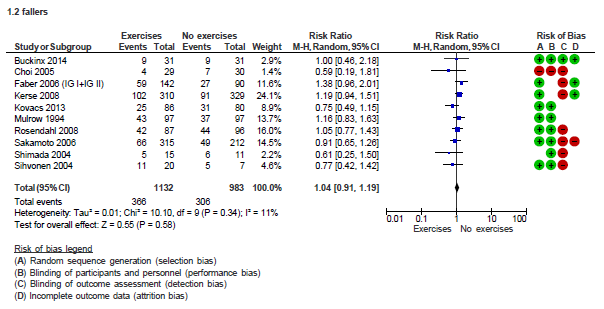


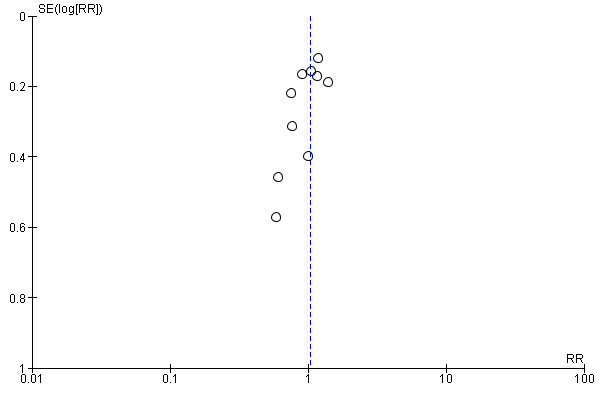


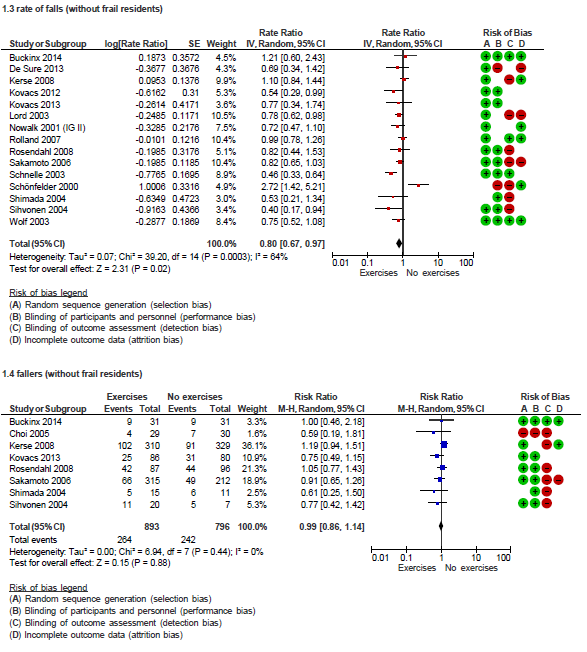


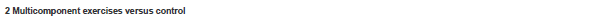


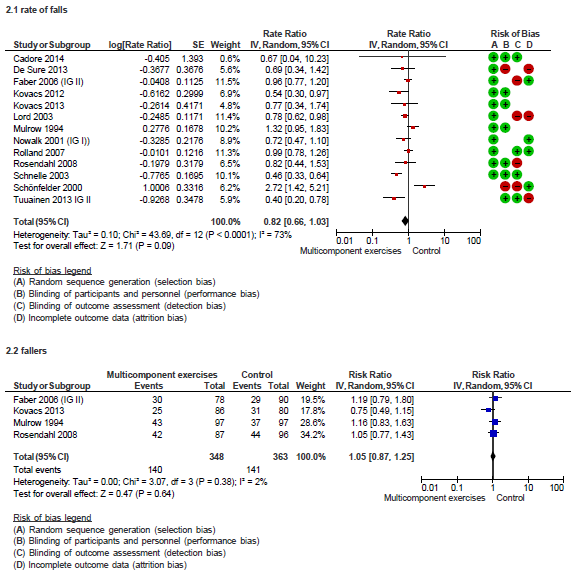


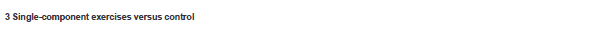

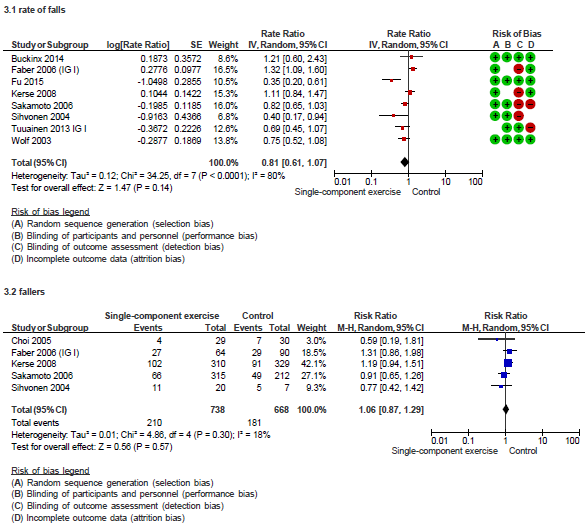


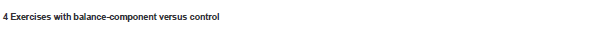


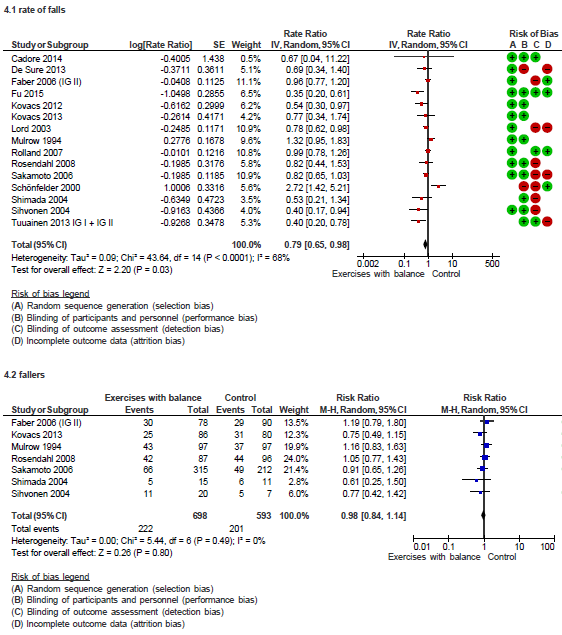


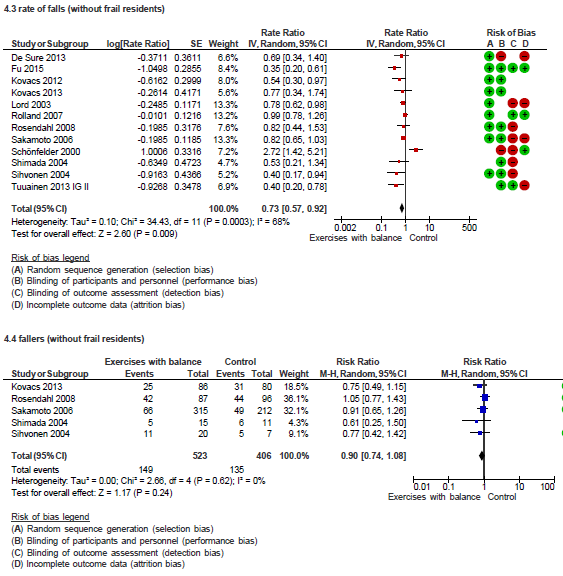


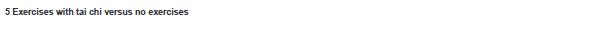


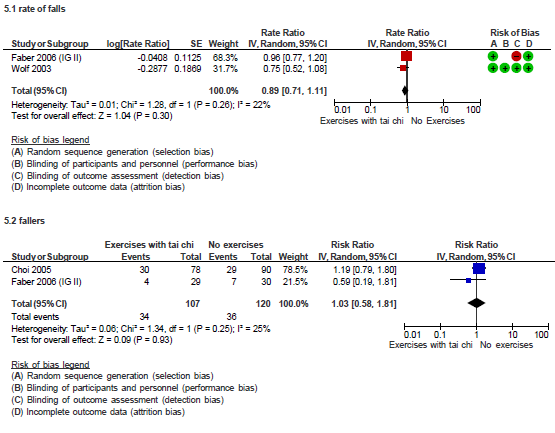


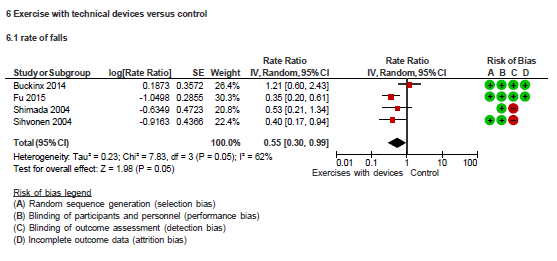

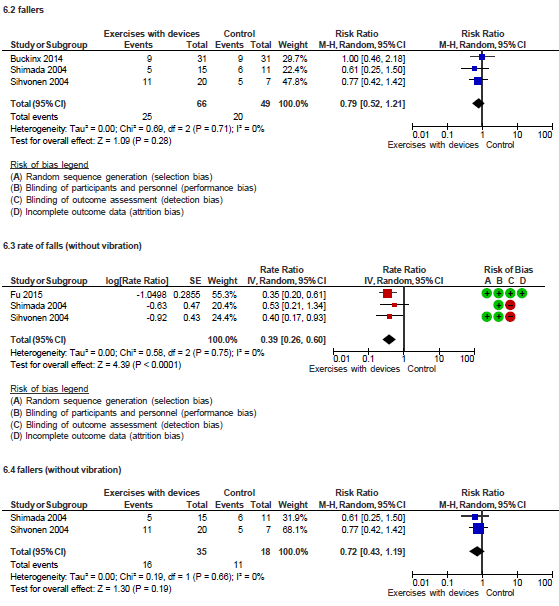


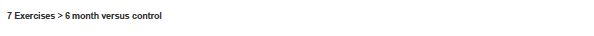


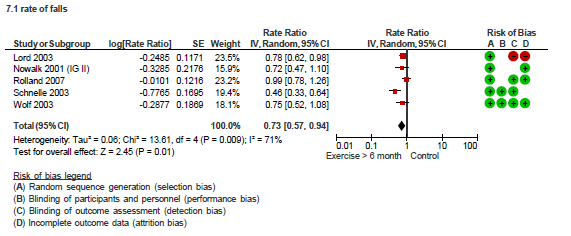


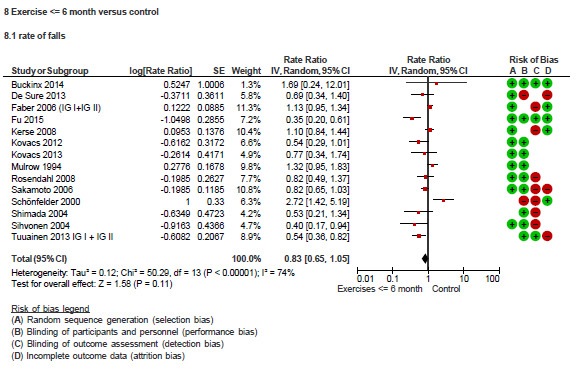

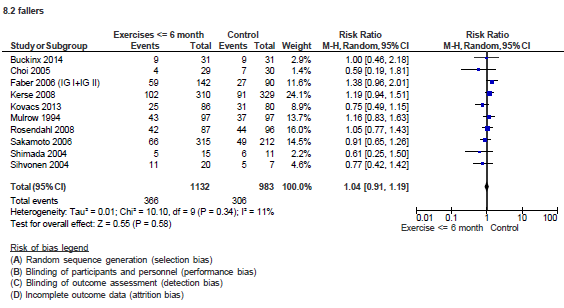


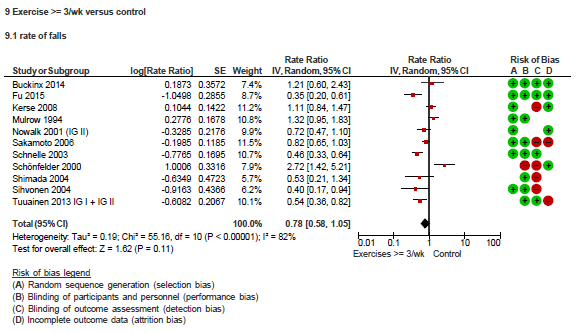


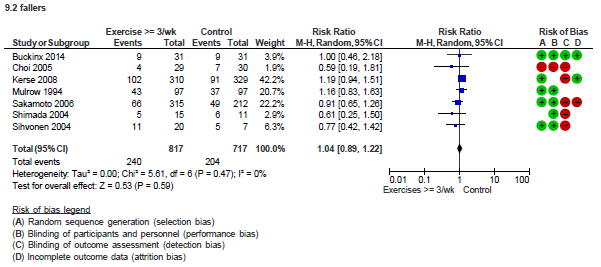


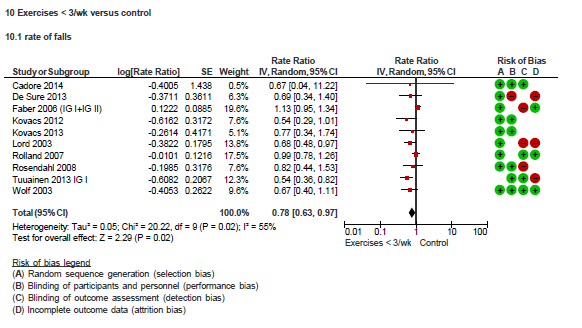


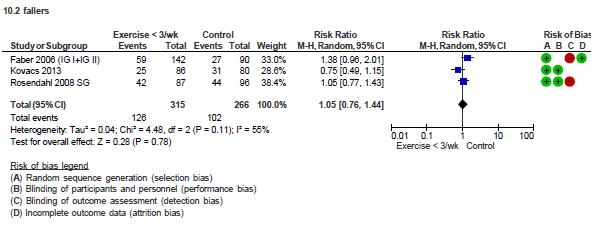


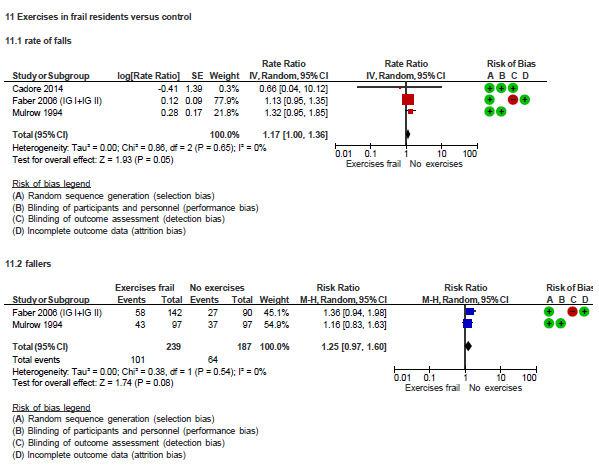


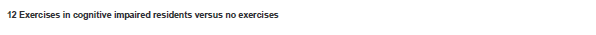


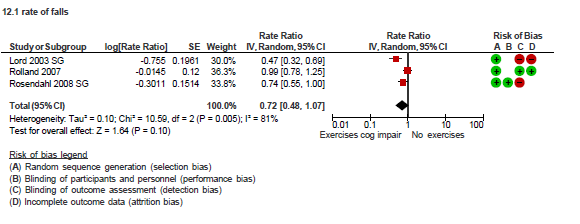

Supplement: Supplementary file 4 [file JAN-76-121-s004.docx]
