# Supplementary material for: Meta‐analysis and GRADE profiles of exercise interventions for falls prevention in long‐term care facilities
Source: J Adv Nurs. 2019 Nov 8;76(1):121–34. doi: 10.1111/jan.14238 (PMC6972676; doi:10.1111/jan.14238)
Supplement: Supplementary file 5 [file JAN-76-121-s005.docx]

**Supplement 5: Forrest Plots of sensitivity analyses (≥ 2 high risk of bias, > studies)**


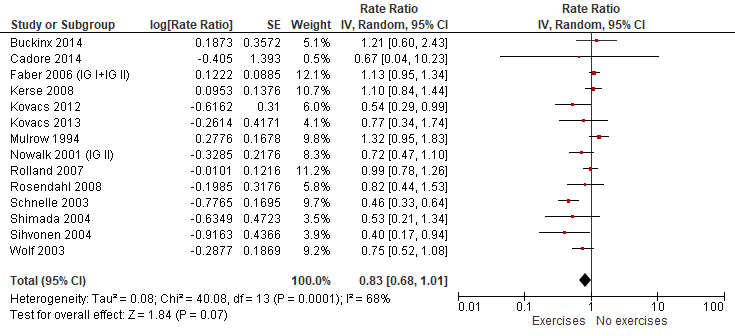


Figure 1: Exercises (any kind of) versus no exercises, outcome: rate of falls (sensitivity analysis)


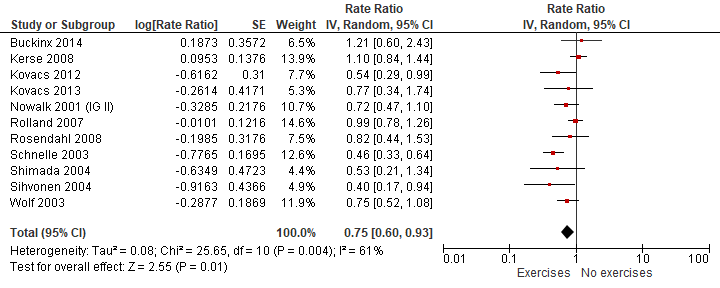


Figure 2: Exercises (any kind of) versus no exercise (without frail residents), outcome: rate of falls (sensitivity analysis)


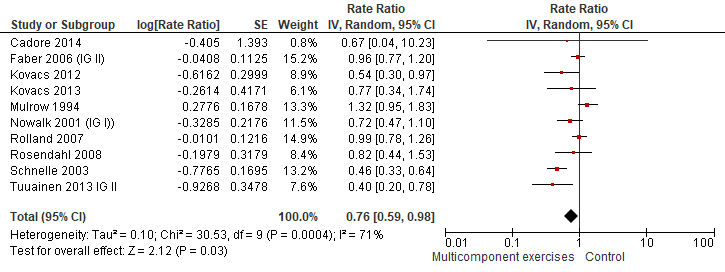


Figure 3: Multicomponent exercises versus control, outcome: rate of falls (sensitivity analysis)


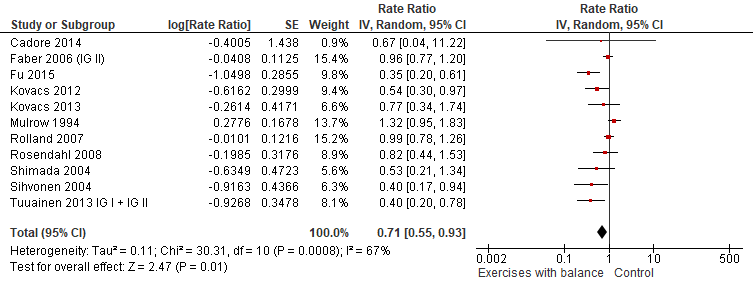


Figure 4: Exercises with balance-component versus control, outcome: rate of falls (sensitivity analysis)


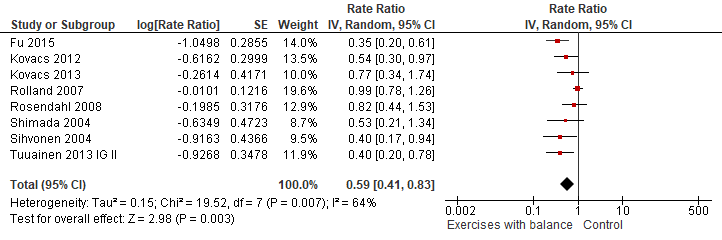


Figure 5: Exercises with balance-component versus control (without frail residents), outcome: rate of falls (sensitivity analysis)


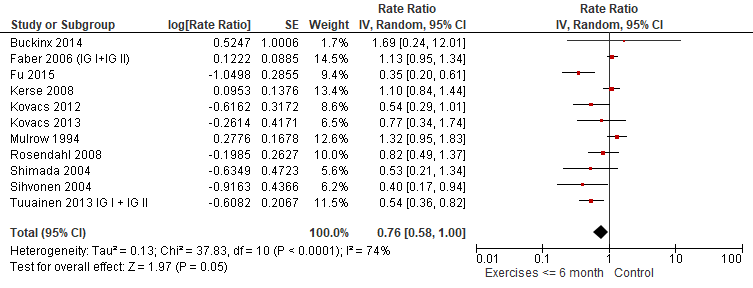


Figure 5: Exercises <= 6 month versus control, outcome: rate of falls (sensitivity analysis)
